# Polypoidal Choroidal Vasculopathy in Asians

**DOI:** 10.3390/jcm4050782

**Published:** 2015-04-24

**Authors:** Chee Wai Wong, Tien Y. Wong, Chui Ming Gemmy Cheung

**Affiliations:** 1 Singapore Eye Research Institute, Singapore National Eye Centre, 11 Third Hospital Avenue, 168751 Singapore, Singapore; E-Mails: wongcheewai81@gmail.com (C.W.W.); gemmy.cheung.c.m@snec.com.sg (C.M.G.C.); 2 Ophthalmology and Visual Sciences Academic Clinical Program, Duke-NUS Graduate Medical School, National University of Singapore, 169857 Singapore, Singapore

**Keywords:** polypoidal choroidal vasculopathy, Asians, epidemiology, risk factors, genetics, photodynamic therapy, anti vascular endothelial growth factor

## Abstract

Age related macular degeneration (AMD) in Asians has been suggested to differ from their Western counterparts in terms of epidemiology, pathogenesis, clinical presentation and treatment. In particular, polypoidal choroidal vasculopathy (PCV) appears to be the predominant subtype of exudative AMD in Asian populations, in contrast to choroidal neovascularization secondary to AMD (CNV-AMD) in Western populations. Epidemiological data on PCV has been largely limited to hospital-based studies and there are currently no data on the incidence of PCV. Similarities and differences in risk factor profile between PCV and CNV-AMD point to some shared pathogenic mechanisms but also differential underlying mechanisms leading to the development of each phenotype. Serum biomarkers such as CRP, homocysteine and matrix metalloproteinases suggest underlying inflammation, atherosclerosis and deranged extracellular matrix metabolism as possible pathogenic mechanisms. In addition, recent advances in genome sequencing have revealed differences in genetic determinants of each subtype. While the standard of care for CNV-AMD is anti-vascular endothelial growth factor (VEGF) therapy, photodynamic therapy (PDT) has been the mainstay of treatment for PCV, although long-term visual prognosis remains unsatisfactory. The optimal treatment for PCV requires further clarification, particularly with different types of anti-VEGF agents and possible benefits of reduced fluence PDT.

## 1. Introduction

Age-related macular degeneration (AMD) is a disease characterized by chronic and progressive degenerative changes in the central retina and is a leading cause of vision loss [1]. The epidemiology, natural history, risk factors, clinical features and treatment of AMD have been well described in Western societies, largely in Caucasian populations [2,3]. AMD has been shown to differ significantly in these areas in Asian populations. In particular, polypoidal choroidal vasculopathy (PCV), a vascular disease of the choroid, appears to be the predominant subtype of exudative or “wet” AMD in Asian populations, in contrast to choroidal neovascularization secondary to AMD (CNV-AMD) in Western populations [4]. There are distinct differences in pathophysiological, clinical and epidemiological factors between the two subtypes, although they also share some common risk factors [5]. In addition, recent advances in genome sequencing have revealed differences in genetic determinants of each subtype. With the advent of anti-vascular endothelial growth factor (anti-VEGF) therapy, patients with CNV-AMD now have an excellent therapeutic option, avoiding what used to be an inexorable progression to central retinal scarring and loss of vision. On the other hand, PCV does not seem to respond as well to anti-VEGF treatment [6]. The optimal treatment option for PCV remains elusive, with most studies showing good short-term visual outcome but poorer longer-term outcome with current treatment strategies. This review summarizes the main advances in our knowledge of PCV in Asians, current therapeutic strategies, outcomes and highlight areas for future research.

## 2. Methodology

We searched MEDLINE using PubMed with the search terms “age-related macular degeneration” or “polypoidal choroidal vasculopathy” in combination with the terms “prevalence”, “incidence”, risk factors”, “gene”, “diagnosis”, “prevention”, “treatment”, “photodynamic therapy” and “anti-vascular endothelial growth factor”. We largely selected publications in the past 5 years, but did not exclude older publications that are commonly referenced or highly regarded. We also searched the reference lists of articles identified by this search strategy and selected those we judged relevant.

## 3. Epidemiology

Age related macular degeneration (AMD) is the third leading cause of blindness in East Asia, and the proportion of blindness attributable to AMD has increased from 5% in 1990 to 6.9% in 2010 [7]. With a global demographic shift towards an aged population, blindness from AMD will present an increasingly significant healthcare problem. This is especially so in Asia, which currently accounts for 60% of the world’s population and will eventually contribute the highest global prevalence of AMD by 2040. In a recent metanalysis of epidemiological studies, Wong *et al*., found an overall global prevalence of 8.69% (95% CrI 4.26–17.40) of any AMD, projected to increase to 288 million affected persons in 2040 [8]. There was no difference in prevalence of neovascular age-related macular degeneration between ethnicities. This has to be taken in the context that PCV is markedly more common in Asians than Europeans and these studies do not differentiate between subtypes of neovascular AMD.

Reports on the incidence of AMD in Asians are rare. Yasuda *et al*., studied the nine-year incidence of AMD in 1775 Japanese subjects ≥40 years old and found a cumulative incidence of 10% and 1.4% for early and late AMD, respectively [9]. You *et al*., found a five-year incidence of 4.2% and 0.1% for early and late AMD, respectively, in the Beijing eye study, a population based study that involved 3049 subjects aged 40–82 years old [10]. Several population-based studies on the prevalence of AMD have been performed in Asia. Prevalence estimates for early AMD and late AMD range from 2.5%–13.8% to 0.2%–7.0%, respectively, with higher prevalence in older study populations [3,11,12,13,14,15,16,17,18,19,20,21,22,23].

Differentiation between PCV and AMD-CNV cannot be made with certainty on fundus photography alone without angiography. As such, accurate estimates of PCV prevalence from population-based studies are difficult to obtain. Most of the known epidemiology of PCV has been derived from hospital or clinic based cross-sectional studies (Table 1). These studies have shown a prevalence of 22.3%–61.6% among Asians [24,25,26,27,28,29,30,31,32] and 8%–13% in Caucasians who present with presumed neovascular AMD [33]. There is a marked male preponderance of 63%–78.5% and only 5.9%–24.1% have bilateral disease. The Beijing Eye Study 2011, a population-based study in Northern China, attempted to estimate the prevalence of PCV using a combined clinical and optical coherence tomography (OCT) criteria to define the presence of PCV. In this study, PCV was defined as an elevated orange-red lesion on fundus photographs, characterized by a double layer sign and high dome-shaped pigment epithelial detachments on the OCT images. With this definition, they found a PCV prevalence of 0.3% ± 0.1%, 95% confidence interval (CI) 0.1%–0.4% [34]. There are currently no incidence estimates for PCV.

**Table 1:** Epidemiological studies of polypoidal choroidal vasculopathy in Asia.

Study	Study Design	Population Size	Race	Age Range	Prevalence of PCV (%)	Male (%)	Bilateral (%)
Li *et al*., 2014 [34] Beijing Eye Study	Population based cross sectional	3468	Chinese	≥50	0.3 ± 0.1,	58.8	5.9
					95% CI 0.1 ± 0.4		
Byeon *et al*., 2008 [30]	Retrospective cross-sectional hospital based	392	Korean	48–85	24.6 *	78.5	24.1
Maruko *et al*., 2007 [29]	Retrospective cross-sectional hospital based	289	Japanese	≥40	54.7 *	77.8	18.4
Coscas G *et al*., 2014 [28]	Retrospective cross-sectional hospital based	99	Japanese	-	48 *	-	-
		94	French	-	9 *	-	-
Mori *et al*., 2010 [32]	Prospective hospital based	373	Japanese	51–96	41.3 *	79.2	13.6
Sho *et al*., 2003 [27]	Retrospective cross-sectional hospital based	471	Japanese	50–93	23 *	63	10
Liu *et al*., 2007 [26]	Prospective cross-sectional hospital based	155	Chinese	50–86	24.5 *	68.4	23.7
Wen *et al*., 2004 [25]	Retrospective cross-sectional hospital based	166	Chinese	≥50	22.3 *	73	13.5
Chang *et al*., 2009 [24]	Retrospective cross-sectional hospital based	100	Taiwanese	-	49 *	71.4	8.2
Cheung *et al*., 2014 [31]	Prospective hospital based	132	Predominantly Chinese	69.5 ± 9.94	61.1 *	58.1	-

* Among patients with presumed neovascular age related macular degeneration.

## 4. Risk Factors

Risk factor analysis provides perspectives on pathogenic mechanisms, uncover potential therapeutic targets, and allow public health-care initiatives to reduce disease burden through preventive medicine. In the case of PCV and CNV-AMD, shared risk factors may imply common pathogenic pathways, while differences in risk factor associations infer differences in their respective pathogenesis. The risk factors for AMD in Caucasian populations have been well described [1]. Age is the most consistent risk factor for both early and late AMD, but may merely reflect the long latent period of the disease. Systemic risk factors that have been associated with increased risk of AMD in Asians include smoking [9,12,19,22,35,36], male gender [11,35], hypertension [12,15,21,35], hyperlipidemia [15], high levels of high density lipoprotein (HDL) [12,21], chronic kidney disease (CKD) [35], Hepatitis B surface antigen (HBsAg) positivity [12], liver cancer [37], coronary heart disease [19] and increased serum white blood cell levels. High levels of serum triglycerides [21], body mass index within the range of 24–28 [19] and alcohol consumption [13] were protective for early AMD while 25-hydroxyvitamin D (in males only) was protective for late AMD [38]. Diabetes mellitus, with or without retinopathy, have been variably associated with both increased [39,40] and decreased risk of AMD [11,37,41].

In the following sections, we focus on similarities and differences in the risk factor profile of PCV and CNV-AMD, and discuss shared/differential serum biomarkers that have been associated with the two diseases.

### 4.1. Systemic Risk Factors

Smoking has consistently been shown to be a risk factor shared by both PCV and CNV-AMD. [5,36,42] Cackett *et al*. showed in their study that persons who smoked were four times more likely to have PCV (OR 4.4 95% CI, 2.5–7.7; *p* < 0.001) or CNV secondary to AMD (OR 4.9 95% CI 2.7–8.8, *p* < 0.001) compared to non-smokers [36]. Similarly, Kikuchi *et al*., found a higher prevalence of smokers with PCV or CNV-AMD compared to controls [42]. Smoking may contribute to the pathogenesis of both diseases through multiple mechanisms, including increased oxidative stress [43], decreased choroidal blood flow leading to ischemia and hypoxia related degenerative changes [44], decreased macular pigment density [45,46], and the promotion of angiogenesis [47]. Patients with PCV and CNV-AMD were also more likely to have diabetes mellitus (DM), hypertension and stroke than control subjects [42].

Some studies have found differential associations between CNV-AMD and PCV with certain systemic risk factors. Sakurada *et al*., compared the associations of systemic risk factors between the two in a hospital-based, cross-sectional, study of 703 cases of PCV and CNV-AMD [40], They found a higher prevalence of DM (OR 2.29 95% CI 1.50–3.52, *p* < 0.001) and end stage renal disease (ESRD) (OR 12.3 95% CI 1.45–104, *p* = 0.021) in patients with CNV-AMD than in PCV. In the same study, no differences were found in the prevalence of hypertension, smoking, stroke and cardiovascular disease between PCV and CNV-AMD. In another study by Ueta *et al*., the prevalence of DM was also found to be higher in CNV-AMD than in PCV (24.7% *vs.* 13.0%; *p* = 0.027) [48], while other systemic risk factors including BMI, hypertension, stroke, ischemic heart disease, hyperlipidemia, smoking and alcohol consumption were similar. Serum levels of vascular endothelial growth factor (VEGF) have been shown to be higher in patients with CKD [49,50], and in uncontrolled diabetic patients with associated microvascular complications, such as nephropathy and retinopathy [51,52], suggesting that VEGF related mechanisms might be more important in the pathogenesis of CNV-AMD than PCV. This theory is supported by previous findings that aqueous levels of VEGF are lower in PCV than in CNV-AMD [53].

### 4.2. Serum Biomarkers

#### 4.2.1. C-Reactive Protein (CRP)

Raised serum levels of CRP, an acute phase protein that rises in response to inflammation, have been inconsistently associated with an increased risk for AMD [42,54,55,56,57,58,59]. However, in a meta-analysis consisting of 11 studies (41,690 study participants) performed by Hong *et al*., high serum levels (>3 mg/L) of CRP were associated with a two-fold likelihood of late AMD (OR 2.189 95% CI 1.379–3.473, *p* = 0.001), compared to low levels (<1 mg/L) [60]. The authors postulate that the reasons for non-association in some studies were a low prevalence of late AMD (and hence lack of statistical power to detect an association) and over-adjustment for inflammatory markers. Of note, Kikuchi *et al*., showed that high CRP levels (>0.95 mg/dL) were associated with a three-fold risk of PCV (OR, 3.53; 95% CI, 1.49–8.40) and a four-fold risk of CNV-AMD (OR, 4.08; 95% CI, 1.94–8.56), after adjusting for age, gender, smoking status, alcohol use, body mass index, history of and use of anti-inflammatory medications [42]. Interestingly, Cheng *et al*. did not find a significant association of PCV with higher CRP levels (OR, 2.45; 95% CI 0.75–7.94, *p* = 0.14), although they did not adjust for the use of anti-inflammatory medications in their study [61]. Taken together, these findings support the hypothesis that inflammation and immune mediated processes are involved in the pathogenesis of both forms of exudative AMD.

#### 4.2.2. Homocysteine

Homocysteine is a non-protein amino acid that is synthesized from methionine. High serum levels of homocysteine can cause endothelial injury, increase oxidative stress and promote thrombosis [62], and has been identified as an independent risk factor for vascular diseases such as stroke [63], coronary heart disease [63], dementia [64] and Alzheimer’s disease [64]. It has also been variably associated with AMD [54,55,65]. In a recent study, Cheng *et al*., found that each 1 µmol/L increase of plasma homocysteine conferred a 1.5-fold increased odds of PCV (OR, 1.54; 95% CI 1.33–1.79, *p* < 0.001). Based on previous histopathological observation of hyalinization in choroidal vessels of eyes with PCV [66], the authors proposed that homocysteine-mediated arteriosclerosis might lead to the development of aneurysmal-like dilations seen in polypoidal lesions.

#### 4.2.3. Matrix Metalloproteinases

Extracellular matrix (ECM) metabolism is regulated by matrix metalloproteinases (MMPs) and tissue metalloproteinase inhibitors (TIMPs). ECM metabolism is a crucial element of vascular remodeling and an imbalance between the MMP/TIMP ratio can result in angiogenesis and pathological changes in vessel wall structure, leading to vascular diseases such as hypertension, abdominal aortic aneurysm, varicose veins and preeclampsia [67]. In PCV and CNV-AMD, histopathological and animal studies have shown that abnormal ECM metabolism may underlie the pathology seen in the bruch’s membrane and the choroidal vasculature [66,68,69]. In particular, the arteriosclerotic and aneurysmal changes seen in PCV strongly suggest derangements in ECM remodeling [66,69]. Chau *et al*., found increased plasma levels of MMP-9 in subjects with early AMD and neovascular AMD, but did not differentiate between PCV and CNV-AMD [70]. Zeng *et al*., further demonstrated, in his pilot study, increased serum levels of MMP-2 and MMP-9 in PCV eyes but not in CNV-AMD or control eyes [71]. With ongoing research undertaken on therapeutics targeting the MMP/TIMP system in tumor angiogenesis, atherosclerosis and aortic aneurysm [67], there is hope that a viable therapeutic agent may also be effective for the treatment of PCV in the near future.

### 4.3. Ocular Risk Factors

Ocular risk factors for early AMD in Asians include hyperopic refraction [10,12,35] and sunlight exposure [15], while smaller optic disc size and shorter scleral spur distance [10] were protective for early AMD. Although cataract surgery has been found to be a risk factor for AMD in Caucasian populations [2], this does not appear to be the case in Asians [72]. Iris pigmentation is another inconsistent risk factor, with some studies in Caucasian populations reporting an association of lighter colored iris with AMD [73]. The ocular risk factors for PCV are relatively unknown. Ueta *et al*. found no difference in cataract surgery, glaucoma or intensive light exposure between patients with PCV and CNV-AMD [48].

#### 4.3.1. Choroidal Thickness

Several studies have demonstrated a markedly thicker choroid in patients with PCV compared to CNV-AMD [34,74,75,76,77]. Chung *et al*. performed enhanced depth imaging (EDI) with the spectral domain OCT (SD-OCT) in eyes with PCV and CNV-AMD, comparing them to age-matched controls. They found significantly greater subfoveal choroidal thickness in eyes with PCV (438.3 ± 87.8 µm, *p* < 0.001), and thinner subfoveal choroidal thickness in eyes with CNV-AMD (171.2 ± 38.5 µm, *p* = 0.004) compared to age matched controls. The authors further propose that choroidal thickness as imaged by EDI OCT may be a useful diagnostic marker differentiating PCV from CNV-AMD [78].

#### 4.3.2. Central Serous Chorioretinopathy (CSCR)

Central serous chorioretinopathy (CSCR) is a disease affecting the central retina, causing exudative retinal detachment with or without a concomitant pigment epithelial detachment. PCV and typical CSCR have distinct phenotypes, but chronic CSCR may mimic PCV and *vice versa* [79,80], in some cases presenting similarly with type 1 neovascularization [81]. This, and other evidence points to a common pathogenesis for both diseases. They share common ICGA findings, such as choroidal vascular hyperpermeability and punctate hyperfluorescent spots [82,83,84,85,86], suggesting that PCV and CSCR may arise from similar choroidal vascular pathology. Similar widespread RPE changes, as seen on fundus autofluorescence imaging, appear in both CSCR [87] and PCV [88]. In addition, several studies have reported that a history of CSCR is a risk factor for PCV development [80,89,90]. Further, studies have demonstrated increased choroidal thickening in both diseases [48,74]. Increased hydrostatic pressure [91] has been proposed as a possible mechanism for the choroidal thickening and hyperpermeability seen in both diseases, but the exact mechanisms leading to the differential development of each phenotype require further elucidation. Interestingly, patients with unilateral PCV or CSC also present with choroidal thickening and vascular hyperpermeability in the unaffected fellow eye, raising the possibility that the choroidopathy seen in both diseases may be part of a systemic vasculopathy [82].

### 4.4. Genetics

To determine if genetic associations with AMD differ between Asians and Caucasian populations, the Genetics of AMD in Asians Consortium performed a genome-wide association study (GWAS) and exome-wide association study (EWAS), enrolling 6345 exudative AMD cases and 15,980 controls from multiple sites in East Asia [92]. Of the 21 loci strongly associated with AMD in European populations [93], nine were replicated in East Asians: *ARMS2- HTRA1* rs10490924, *CFH* rs10737680, *CETP* rs3764261, *ADAMTS9* rs6795735, *C2*-*CFB* rs429608, *CFI* rs4698775, *TGFBR1* rs334353, *APOE* rs4420638, and *VEGFA* rs943080. Four new loci were found, and in particular, *CETP* Asp442Gly (rs2303790), a mutation that is specific to East Asians, was found to be strongly associated with exudative AMD (OR 1.70). Interestingly, several studies have found *CETP* genetic variants to be associated with a high risk of PCV, thus suggesting possible involvement of the high-density lipoprotein metabolism pathway in the pathogenesis of PCV [94,95,96].

Using the candidate gene approach, many studies have examined genetic loci linked to AMD for possible association with PCV. Chen *et al*., performed a systematic review and meta-analysis of published articles on the genetic associations of PCV and found variants in four genes to be significantly associated with PCV [97]. These include two single nucleotide polymorphisms (SNPs) in the complement factor H (*CFH*) gene: rs1061170 (allelic odds ratio (OR) = 1.72, *p <* 0.00001) and rs800292 (*n =* 5, OR = 2.10, *p <* 0.00001), and two in the *ARMS2/HTRA1* loci: LOC387715 rs10490924 (OR=2.27, *p <* 0.00001) and rs11200638 (OR = 2.72, *p <* 0.00001). Another complement gene variant, *C2* rs547154 (OR = 0.56, *p* = 0.01) was found to be protective for PCV. The same study analyzed seven other genes that were previously reported to be associated with PCV (*CFB*, *C2*, *SERPING1*, elastin, *SOD2*, *PEDF*, *TLR3*, and *9p21*) but did not find significant association. Interestingly, the ARMS2 *LOC387715* rs10490924 was the only variant showing a significantly weaker association with PCV than with CNV-AMD (OR = 0.66, *p <* 0.00001), suggesting that genetic differences could guide the differential development of each phenotype. This variant was also associated with larger lesion size, higher likelihood of vitreous hemorrhage, and worse visual outcome one year after treatment with PDT or combination therapy in PCV. Gotoh *et al*., too found larger lesion size to be associated with a SNP in *HTRA1* (rs11200638), but there were no significant difference in the incidence of *CFH* rs1061170 or *HTRA* rs11200638 between eyes with PCV or CNV-AMD [98]. The associations with PCV of two of these genetic variants, CFH I62V polymorphism (rs800292) [99] and ARMS2 A69S (rs10490924) [100] polymorphism were replicated in recent meta-analyses. Tanaka *et al*. further demonstrated that two distinct phenotypes of PCV, typical PCV and polypoidal PCV, were differentially associated with *CFH I62V* and *ARMS2 A69S*, respectively [101].

To date, only one genome wide association study (GWAS) has been performed for PCV, in part due to the difficulty of diagnosing PCV. This was performed by Goto *et al*. in Japanese patients with PCV (*n =* 100); 100 with CNV-AMD (*n =* 100) and 190 age-matched controls. Three single nucleotide polymorphisms (SNPs) reached GWAS significance in this study: *ARMS2/HTRA1* rs10490924 (OR = 2.72, *p* = 3.7 × 10^−8^), *CFH* rs800292 (OR = 2.00 *p* = 2.6 × 10^−4^), and *C3* rs2241394 (OR = 3.47 *p* = 2.5 × 10^−3^) [102]. Given the distinct phenotypic differences between PCV and CNV-AMD, it is likely that genes not previously associated with AMD could be associated with PCV. Future GWAS studies with larger sample sizes and replication cohorts in populations of different ethnicities may help clarify the differences in genetic basis of the two diseases.

#### Pharmacogenetics

In terms of pharmacogenetic biomarkers of response to PDT in PCV, *ARMS2 A69S* (rs10490924) has been shown to predict worse visual outcome at 12 months and more frequent recurrence [103], while pigment epithelium derived factor gene polymorphism (*SERPINF1* rs12603825) was associated with a shorter retreatment-free period after initial PDT and had significantly worse visual outcome [104]. As for response to anti-VEGF, Yamashiro *et al*. found no significant influence on outcomes with intravitreal ranibizumab by genetic polymorphisms at *CFH* and *ARMS2/HTRA1* [105]. Another study examining the combination therapy of PDT with intravitreal bevacizumab injection in PCV of Koreans found that the risk genotypes, TT of rs10490924 and AA of rs11200638 at *ARMS2/HTRA1*, had significantly poorer outcome after one year of follow-up [106]. On average, subjects with the TT genotype at rs10490924 had significantly less absence of leakage (*p* = 0.04), less polyp regression (*p* = 0.006), and worse visual acuity (*p* = 0.034) at 12-month follow-up. This was similar for the AA genotype at rs11200638, with significantly less absence of leakage (*p =* 0.019), less polyp regression (*p =* 0.002), and worse visual acuity (*p =* 0.022) at 12-month follow-up. The intermediate risk phenotypes also had an intermediate outcome after combined PDT and bevacizumab injection.

## 5. Clinical Features and Natural History

**Figure 1:**
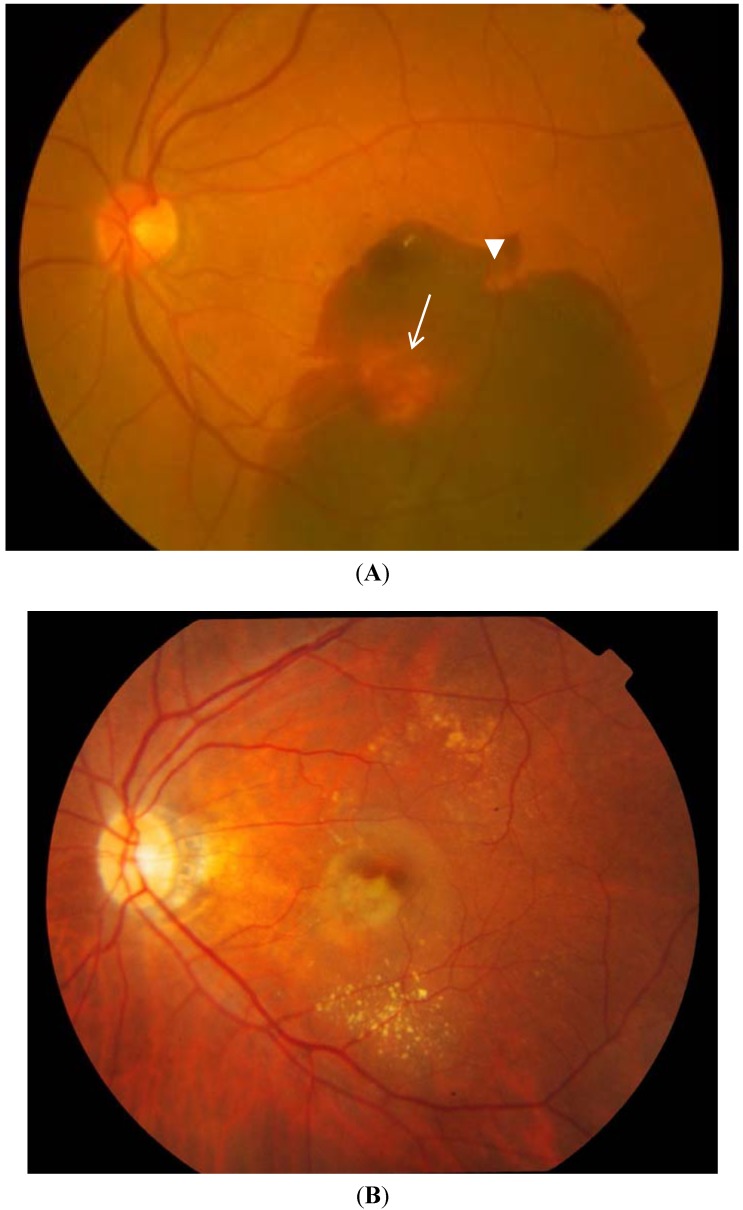
Fundus photographs showing the two clinical patterns of polypoidal choroidal vasculopathy: Hemorrhagic (**A**) and exudative (**B**). An orange red nodule suggestive of a polyp (white arrow) is seen in an extrafoveal position, while a notch in the hemorrhagic pigment epithelial detachment (white arrowhead) suggests the presence of another polyp.

Clinically, PCV may present with polypoidal lesions visible as orange-red nodules in the macular or peripapillary region, often associated with serosanguinous PEDs, but without associated drusen [27,107,108,109]. When a large PED is present, a notch in its margin frequently indicates the site of polypoidal lesions [102]. Uyama *et al*. described the natural history of untreated PCV in 14 eyes over 24–54 months. PCV presented in two patterns: exudative, characterized by serous PED and retinal detachment, or hemorrhagic, characterized by hemorrhagic PED and subretinal hemorrhage at the macula (Figure 1) [110]. Half of these patients had a favorable course, while the other half had recurrences and eventual visual loss. Cheung *et al*. studied 32 untreated eyes with PCV over a mean follow up of five years and similarly observed that half of them had visual deterioration due to hemorrhage and scarring. Unlike Uyama *et al*., who found that patients with a cluster of grapes configuration of polypoidal lesions on ICGA had worse visual outcome, none of the presenting features were found to influence the visual outcome significantly [111].

## 6. Angiography

**Figure 2:**
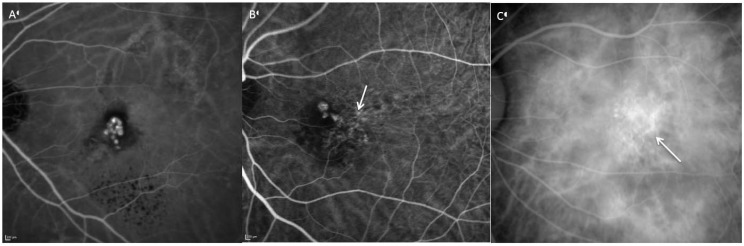
Angiographic patterns of PCV: Cluster of grapes configuration (**A**), solitary (**B**) and string of pearls (**C**). Branching vascular networks (white arrow) are seen in figure B and C.

Because PCV is primarily a pathology of the choroidal vasculature, indocyanine green is the angiographic dye of choice for diagnosis as it has higher protein binding affinity and does not leak from the choriocapillaris like fluorescein. In addition, indocyanine green emits near infra-red light, which penetrates the RPE more readily than the green light emitted by fluorescein [112]. Indocyanine green angiography (ICGA) is considered the gold standard for diagnosis of PCV and is essential for distinguishing between CNV-AMD, retinal angiomatous proliferation (RAP) and PCV. Currently, there is no universally accepted ICGA definition of PCV, with most studies using a combination of clinical and ICGA features. The Japanese Study Group of Polypoidal Choroidal Vasculopathy defined definite cases as protruded orange-red elevated lesions on fundus examination and/or characteristic polypoidal lesions on ICGA. Probable PCV was diagnosed if there was only an abnormal vascular network or occurrence of recurrent hemorrhagic and/or serous detachments of the retinal pigment epithelium were observed [113]. In the EVEREST study, the diagnosis of PCV was made on the basis of subretinal focal ICGA hyperfluoresence in the presence of at least one of the following: branching vascular network (BVN); pulsatile polyp; nodular appearance on stereoscopic viewing; hypofuorescent halo; orange subretinal nodule on color fundus photo; and presence of massive submacular hemorrhage [114]. Polypoidal lesions most commonly appear in clusters, but could occur in isolation or in a string configuration on ICGA (Figure 2) [115]. Even with well-defined criteria, diagnosis of PCV is not straightforward because of the complexity of angiographic findings and overlap of signs with CNV-AMD. The concordance of PCV and CNV-AMD diagnoses was 77% even among retinal specialists experienced in the management of PCV, highlighting the need for clearer diagnostic criteria [28]. An additional consideration is the method of capturing ICGA images, which can be done with either a flash photography system or a confocal scanning laser ophthalmoscope (cSLO). In the study by Cheung *et al*., comparing the two systems, typical nodular appearance was the most commonly detected feature, with the highest area under the curve for diagnosis of PCV and both systems were similarly sensitive in picking up this sign. However, cSLO was found to be more sensitive in detecting BVN and late hyperfluoresecent plaques, additional features that will aid the diagnosis of PCV especially when nodular lesions were not present [116]. Angiographic features of PCV may serve as prognostic factors as well. These include: (1) clusters of grapes configuration of polyps [110,117,118]; (2) type of abnormal vascular network. Polyps supplied by interconnecting channels were found to have the best visual outcome following treatment, followed by PCV with non-leaking BVN while PCV with leaking BVN had the worst visual outcome [119]; (3) presence of choroidal vascular hyperpermeability may predispose to poor response with intravitreal ranibizumab [90,120,121]; (4) patients with pulsating PCV on ICG video angiography had a higher risk of extensive hemorrhagic events [122]; and (5) larger PCV lesions. Tsujikawa *et al*., found that, compared to smaller PCV lesions, larger PCV lesions were more likely to progress in lesion size, had a higher risk of complications including RPE tears, vitreous and suprachoroidal hemorrhage, and were associated with poorer visual outcome [123].

## 7. Fundus Autofluorescence

**Figure 3:**
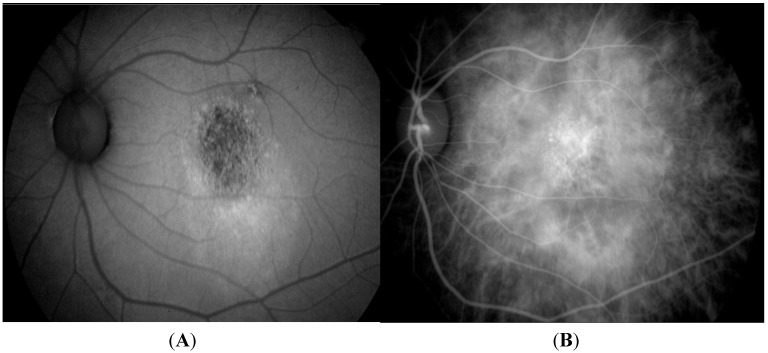
Fundal autoflorescence image (**A**) showing granular hypoautofluorescence, which corresponds to the abnormal branching vascular network seen on indocyanine green angiography (**B**).

Yamagishi *et al*., described two characteristic fundus autofluoresence (FAF) patterns in eyes with PCV: Confluent hypoautofluoresence and granular hyperautofluorescence, each corresponding to different components of the PCV lesion (Figure 3) [88]. Confluent hypoautofluorescence, mostly associated with a surrounding hyperautofluorescent ring, correspond to polypoidal lesions seen on ICGA in 80.4% of eyes with PCV. This pattern is not seen in patients with CNV-AMD. Granular hyperautofluorescence corresponds to the branching choroidal vascular network, and is seen somewhat more frequently in eyes with PCV than in eyes with CNV-AMD (98.9% *vs*. 87.1%, *p =* 0.014). Lastly, the unaffected fellow eyes of patients with PCV present more frequently with hyperautofluorescence, both within (*p =* 0.012) and outside the macula (*p =* 0.003), than eyes with CNV-AMD. This finding is in agreement with a previous study by Ueta *et al*., that showed widespread and bilateral damage to the RPE occur more often in patients with PCV than in patients with CNV-AMD [124]. Yamashigi further demonstrated that the hyperautofluorescent ring disappeared in 71.4% of patients with resolved polyps at one-year post treatment, and suggested the use of FAF as a useful, non-invasive adjunct to ICGA and OCT for diagnosis and monitoring of treatment. With well-defined diagnostic criteria and in conjunction with other non-invasive imaging techniques, FAF may be a valuable diagnostic tool for PCV, particularly in population based epidemiologic studies and in patients allergic to indocyanine green. In a longer term study of FAF changes after treatment of PCV, Suzuki *et al*., found an increase in size of granular hyperautofluorescence, associated with a drop in visual acuity, highlighting the importance of treating abnormal branching vascular networks in patients with PCV to prevent progressive damage to the overlying RPE [125].

## 8. Optical Coherence Tomography

**Figure 4:**
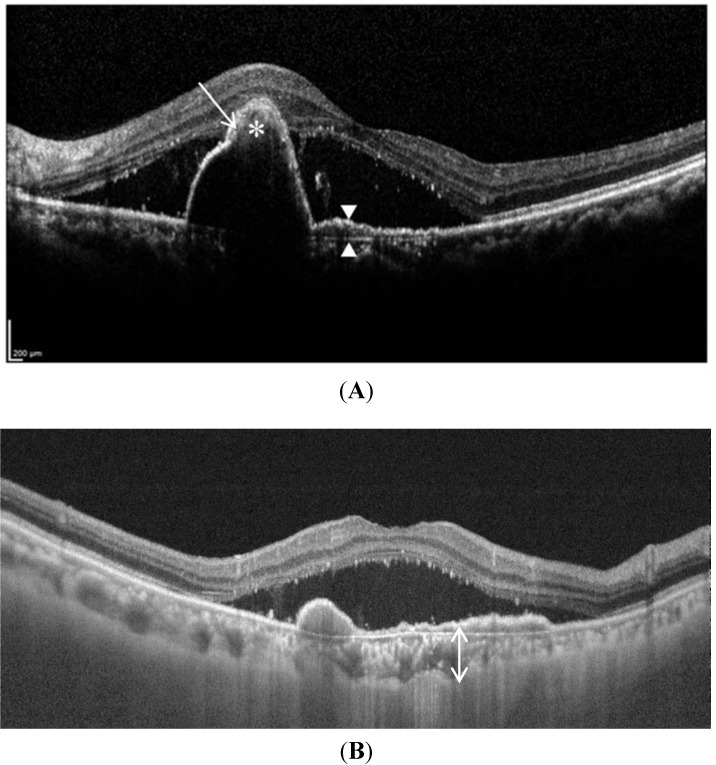
Tomographic features of polypoidal choroidal vasculopathy (PCV): Spectral domain optical coherence tomography (OCT) (**A**) shows peak like elevation of the RPE with underlying moderate reflectivity within the peak (asterisk), tomographic notch in the PED (white arrow) and double layer sign, consisting of two hyper-reflective lines (white arrowheads). Swept source OCT (**B**) is able to visualize the underlying thickened choroid (double headed arrow).

Optical coherence tomography (OCT) works on the principle of interferometry, capturing light reflected from the plane of interest to produce high-resolution images, enabling near histologic visualization of the anatomical features of PCV. Various OCT features of PCV have been reported (Figure 4): (1) peak like elevation of the RPE with underlying moderate reflectivity within the peak [126,127], most likely representing the polyp itself; (2) a notch in the PED, representing a polypoidal lesion at the margin of the PED [102]; and (3) the double layer sign, consisting of two hyper-reflective lines, representing the RPE and Bruch’s membrane, respectively, and corresponding to the extent of late geographic hyperfluorescence on ICGA. This feature is thought to originate from fibrous tissue harbored by the branching vascular network [128,129].

**Figure 5:**
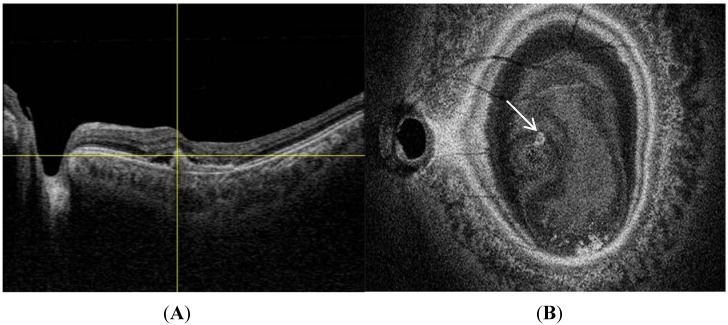
Enface swept source optical coherence tomography (OCT) image (**B**) shows the position of a polyp (white arrow) that corresponds in location to the polyp (intersection of yellow lines) seen on swept source B, scan image (**A**).

Because of the level of detail provided by OCT, there is hope that this imaging modality may provide sufficient diagnostic sensitivity and specificity to replace ICGA and provide a fast, non-invasive way to diagnose PCV. To this end, De Salvo *et al*., compared the use of SD-OCT and ICGA to diagnose PCV and differentiate from occult CNV, based on the following tomographic findings: sharp PED peak, PED notch, hyporeflective lumen within hyperreflective lesions adherent to retinal pigment epithelium. They found a sensitivity of 94.6%, specificity of 92.9%, positive predictive value of 97.2% and a negative predictive value of 86.7% for diagnosing PCV [130]. The lower negative predictive value suggests that better clarification of the tomographic features of PCV may improve the diagnostic utility of OCT. This may potentially be achieved through advances in OCT technology, namely the evolution to high penetration OCT (HPOCT)/swept source OCT (SSOCT), which has allowed improved visualization of the sub-RPE features of PCV [131,132,133]. In a recent study, Sayanagi demonstrated that HPOCT may, in fact, offer almost similar visualization of polyps (Figure 4) and abnormal choroidal vasculature as ICGA [134]. With HPOCT en face images of the RPE layer, they observed RPE rings with inner reflectivity that corresponded to 95% of polypoidal lesions detected on ICGA (Figure 5). These RPE rings were either the same size as polypoidal lesions on ICGA, or larger but corresponded in size with the hypofluorescence around the polyp. In 47% of these RPE rings, the underlying dilated choroidal vessels could be seen, compared to 33% in polypoidal lesions seen on ICGA. Abnormal choroidal vascular networks were seen as a highly reflective mesh-like configuration on HPOCT en face images. Future studies using a comprehensive set of tomographic criteria to evaluate the diagnostic utility of HPOCT/SSOCT may pave the way to non-invasive and accurate diagnosis of PCV.

## 9. Other Imaging Modalities

### 9.1. Optical Coherence Doppler Angiography

FA and ICGA are necessary investigations for exudative AMD but they have several inherent limitations. First, they are invasive methods requiring painful intravenous cannulation; second, the injected dye may induce adverse reactions and may be contraindicated in some patients; and third, it is expensive and repetitive examinations may not be practical. Although structural visualization offered by conventional OCT alone cannot replace the functional information provided by ICGA, this may soon be changed with the emergence of optical coherence doppler angiography. The Doppler effect, referring to the change in frequency of a wave with movement of an observer relative to the source, coupled with ultrasound technology (Doppler ultrasound), has been utilized in the non-invasive imaging of deep vasculature elsewhere in the body. Similarly, angiography of the retinal and choroidal vasculature may be possible with Doppler OCT. Doppler OCT detects the doppler shift induced from red blood cells flowing in vessels to selectively image the vasculature. By combining high penetration OCT with optical coherence angiography (HPOCA), it may now be possible to image deep choroidal vasculature [135]. A prototype device has been built by the University of Tsukuba and shown to be able to visualize feeder vessels in PCV and provide similar but higher contrast angiograms than ICGA mid phase angiograms [136]. Larger studies with HPOCA are needed to determine its utility in clinical practice compared to conventional angiography.

### 9.2. Retro-Mode Imaging

Retro-mode imaging with the confocal scanning laser ophthalmoloscope (cSLO) has recently been investigated as a potential non-invasive test for PCV diagnosis [137,138]. The cSLO normally collects directly backscattered light from the confocal plane through a confocal aperture. Deviating the aperture laterally and using an annular aperture forms a shadow to one side of an abnormal feature, enhancing its contrast. Zeng *et al*. evaluated this imaging modality in 29 eyes with PCV and found that 93.1% of eyes with polyps were clearly imaged, and detected BVN in 55.2%. They found no difference between retro-mode imaging and ICGA for finding polyps and/or BVN [137]. However, retro-mode imaging is unable to detect disease activity and is not able to localize the depth of the lesion, thereby limiting it to a complementary role.

## 10. Treatment

Regarding PCV, treatment options include laser photocoagulation, PDT, anti-VEGF therapy, or a combination of these modalities. The optimal management of PCV remains elusive, mainly due to a lack of high quality randomized controlled trials.

### 10.1. Laser Photocoagulation

The use of laser photocoagulation for CNV-AMD was first established by the Macular Photocoagulation Study Group [139,140,141,142,143], but has largely been limited to extrafoveal and juxtafoveal lesions. In recent years, thermal laser photocoagulation had been superceded by the superior efficacy of photodynamic therapy and subsequently anti-VEGF agents and concerns about the risk of decreased visual acuity from laser scar formation or recurrent CNV when applied close to the fovea. However, thermal laser still has a role in the treatment of PCV because unlike CNV-AMD, a substantial proportion of PCV lesions arise extrafoveally [115,144], and while PDT has proven efficacy in the treatment of PCV, it is expensive, not always accessible to all patients and there are concerns regarding hemorrhagic complications and choroidal hypoperfusion after PDT [145,146,147,148]. Some important questions remain regarding the use of laser photocoagulation for PCV. (1) Is it effective? A few small retrospective studies have shown promising success rates, achieving stable or improved visual acuity in 56%–78% of treated eyes [108,149,150,151,152], but there is a lack of large controlled randomized trials to provide conclusive evidence. (2) Should treatment be applied to polypoidal lesions alone or should the associated abnormal vascular network be included as well? Abnormal vascular networks are believed to be responsible for persistent exudation and poor visual outcome after treatment of polypoidal lesions alone. To answer this question, Yuzawa *et al*., performed a retrospective comparison of 47 eyes with PCV treated with laser photocoagulation to either polyps alone or polyps and the associated abnormal vascular network (whole lesion). Eyes that received treatment of whole lesions achieved 90% stabilization or improvement of visual acuity and resolution of exudation and/or blood after one treatment, while only 46% of eyes with treatment to polyps alone achieved stabilization or improvement of visual acuity [153]. The authors concluded that treatment of whole lesions is recommended, but a few problems remain: abnormal vascular networks may span a large area, necessitating the application of excessive laser photocoagulation, and the borders of these networks are often indistinct or obscured by hemorrhage. (3) Can adjunctive treatment with anti-VEGF agents improve outcomes? Foveal involvement from exudation or hemorrhage secondary to extrafoveal PCV is not uncommon [144] and adjunctive anti-VEGF treatment may be beneficial in the subset of cases with persistent exudation after polyp closure with thermal laser. Cheung *et al*., performed a prospective study of eyes with extrafoveal PCV treated with thermal laser, with or without adjunctive anti-VEGF given at the discretion of the treating clinician [144]. Stable or improved vision was achieved in 90.3% of cases, higher than any previous studies of thermal laser treatment for extrafoveal PCV. There were no significant differences in visual outcome between eyes treated with or without adjunctive anti-VEGF in this study, although eyes that received anti-VEGF had thicker central subfield thickness at baseline with resolution of this difference at three months post treatment. Thus, adjunct anti-VEGF treatment appears to be beneficial at least in patients with significant central retinal thickening but further research is needed to optimize the selection of cases for combination treatment. In summary, laser photocoagulation and adjunctive anti-VEGF treatment may be a viable and less costly alternative for patients with central retinal thickening resulting from extrafoveal PCV.

### 10.2. Photodynamic Therapy

The EVEREST study [114] was the first randomized controlled trial evaluating standard fluence PDT with or without ranibizumab 0.5 mg and ranibizumab monotherapy. This double masked, placebo controlled trial randomized 61 Asian patients into three groups: PDT monotherapy, ranibizumab monotherapy and combination therapy with PDT and ranibizumab. The primary endpoint was the proportion of patients with ICGA confirmed complete regression of polyps at six months. The authors found a higher polyp closure rate of PDT with or without ranibizumab compared to ranibizumab alone (77.8% and 71.4% *vs*. 28.6%; *p <* 0.01). This study established the efficacy of PDT in the closure of PCV polyps, but was not adequately powered to assess differences in visual acuity or central retinal thickness changes. To assess the effect of PDT *versus* anti-VEGF in terms of visual outcome, the LAPTOP study, a multicentered randomized controlled trial was conducted [146]. Ninety-three patients were randomized to two arms: standard fluence PDT monotherapy arm and a ranibizumab monotherapy arm where patients received three monthly injections of ranibizumab 0.5 mg. Additional treatment was performed as needed in each arm. At 12 months, the study found a higher proportion of patients gaining more than 0.2 logMAR units in the ranibizumab arm (30.4% *vs*. 17.0%, *p =* 0.039). In addition, the mean gain in logMAR visual acuity was also greater in the ranibizumab arm at 12 months (*p =* 0.011) [146] and at 24 months (*p =* 0.025) [154]. These two trials showed that although PDT may be more effective at polyp closure than anti-VEGF, anti-VEGF therapy seemed to be better for improving or preventing visual loss in patients with PCV.

### 10.3. Anti-Vascular Endothelial Growth Factor Therapy

In addition to the above-mentioned EVEREST and LAPTOP trials, where ranibizumab monotherapy was studied as one of the treatment arms, many studies have assessed the efficacy of anti-VEGF monotherapy in treatment naïve eyes with PCV. Table 2 shows a selection of these studies with follow up of six months or more [155,156,157,158,159,160,161,162]. The anti-VEGF regime consisted of monthly injections for the first three months, followed by as needed reinjections in most of these studies, except in the PEARL trial [155] where ranibizumab was given monthly. The polyp regression rates achieved by ranibizumab monotherapy at six months ranged from 25% to 33%, comparable to that in the EVEREST trial. With regards to longer-term efficacy, Hikichi *et al*., found a polyp regression of 40% at 12 months, which dropped to 25% at 24 months. Abnormal choroidal vascular complexes do not appear to be affected by anti-VEGF monotherapy [155,156]. The proportion of eyes with ≥0.3 logMAR BCVA improvement ranged from 17%–33.3% [155,157,158] and 40% [159,161] at six and 12 months, respectively. Regarding prognostic factors for visual outcome after ranibizumab monotherapy, Kang *et al*., found that smaller lesion size, absence of PED at baseline and no recurrences predicted better visual outcome [156], while Koizumi *et al*., found smaller lesion size to be an independent predictor for resolution of subretinal fluid one month after three monthly injections of ranibizumab [163].

There are fewer studies assessing the efficacy of bevacizumab, and most of them have short follow up duration or were not performed on treatment naïve patients [164,165,166,167,168,169,170,171]. In a retrospective case control study, Cho *et al*. compared ranibizumab with bevacizumab monotherapy and found no difference in polyp regression rate, central macular thickness and visual acuity at six months [158]. Larger series with longer follow up and head to head comparative trials with PDT/ranibizumab are needed to determine the efficacy of bevacizumab in the treatment of PCV.

Aflibercept (Eylea; Bayer HealthCare, Berlin, Germany) is a recombinant fusion protein designed for improved binding affinity (about 140 times that of ranibizumab) to VEGF receptors A, B and placental growth factor. A few studies have investigated the efficacy of aflibercept in the treatment of PCV. In a small, non-comparative prospective study of 16 treatment naïve eyes with PCV, Inoue *et al*., found a high rate of polyp regression (75%) at six months with aflibercept monotherapy given at two monthly intervals after three initial monthly loading doses [172]. This is in contrast to the low polyp regression rate achieved by ranibizumab monotherapy in other studies (28.6%–40%), the EVEREST trial (28.6%) [114] and the PEARL study (33%) [155]. However, abnormal branching vascular networks persisted in all eyes, representing a risk for recurrence, a result shared by ranibizumab monotherapy [114,155,161]. Ljiri *et al*., on the other hand, found a lower polyp regression rate (48%) with the same regimen of aflibercept in a prospective series of 33 treatment naïve eyes, albeit at three months after initiation of treatment [173]. Oishi *et al*. compared the efficacy of aflibercept in CNV-AMD, PCV and RAP. He observed a 69% polyp closure rate at one year after treatment with aflibercept and no difference in visual improvement between CNV-AMD and PCV, contradicting previous studies that suggested anti-VEGF therapy was less effective in PCV compared to CNV-AMD. In the same study, the authors found that the predictive factors for good visual outcome were the presence of polypoidal lesions, external limiting membrane (ELM) and a smaller greatest linear dimension (GLD) [174]. Neovascular AMD with persistent disease activity refractory to ranibizumab monotherapy appears to respond well to a switch in therapy to aflibercept [175,176,177,178]. The beneficial effect of switching to aflibercept has been shown in eyes with PCV as well [179,180]. In a retrospective study of 43 eyes with PCV refractory to ranibizumab monotherapy, Saito *et al*., demonstrated further improvement in visual acuity (form 20/48 to 20/43), central retinal thickness and a 50% polyp regression rate at three months [180]. The authors proposed several reasons for the improved efficacy: tachyphylaxis to ranibizumab, higher binding affinity to VEGF and promotion of thrombus formation with occlusion of polyps. This study showed yet again a higher polyp closure rate than ranibizumab, suggesting that aflibercept may have superior efficacy. Finally, the Intravitreal Aflibercept Injection (Eylea) for Polypoidal Choridal Vasculopathy with Hemorrhage or Exudation EPIC trial, the first prospective trial evaluating the efficacy of intravitreal aflibercept in the treatment of PCV, found a decrease in retinal pigment epithelial detachment in over 90% of cases at six months (Results presented at EURETINA 2014) [181].

Future studies, including head to head trials with ranibizumab, PDT or as an adjunct to PDT are needed to confirm these findings.

**Table 2:** Anti-VEGF monotherapy for treatment naïve PCV.

Study	Study Design	Sample Size	Follow up (Months)	Anti-VEGF	Mean Number of Injections	Central Macular Thickness	Polyp Regression (%)	BCVA (logMAR)	≥0.3 logMAR BCVA Improvement (%)
Koh *et al*., 2012 [114] EVEREST study	Randomized controlled trial	21	6	Ranibizumab	5	−65.7 ± 114.3 *	28.6	9.2 ± 12.4	33.3
Cho *et at*., 2015 [157]	Retrospective Case control	69	6	Ranibizumab	4.0	-	26.1	0.59	33.3
Kokame *et al*., 2009 [155] PEARL trial	Prospective trial	12	6	Ranibizumab	6	265 ± 204	33	51.0 ETDRS letters	17
Cho *et al*., 2012 [158]	Retrospective case control	58	6	Bevacizumab	3.31 ± 1.25	274 ± 40.77	20.7	0.74 ± 0.51	20.7
		52	6	Ranibizumab	3.44 ± 0.92	286 ± 36.93	25	0.78 ± 0.43	21.2
Oishi *et al*., 2013 [146,154] LAPTOP study	Randomized controlled trial	46	12	Ranibizumab	4.5	311.2 ± 146.9	-	0.39 ± 0.26	17.4
		46	24		1.4	291.2 ± 129.3	-	0.40 ± 0.37	28.3
Hikichi *et al*., 2013 [182]	Prospective	75	12	Ranibizumab	4.2 ± 1.3	211 ± 45	40	0.37 ± 0.33	37
			24		1.6 ± 1.7	213 ± 42	25	0.41 ± 0.40	33
Ogino *et al*., 2013 [160]	Prospective	23	12	Ranibizumab	6.1 ± 2.8	175 ± 70	-	0.30 ± 0.34	-
Hikichi *et al*., 2012 [161]	Retrospective	85	12	Ranibizumab	4.2 ± 1.3	211 ± 10	40	0.37 ± 0.30	37
Matsumiya *et al*., 2013 [162]	Retrospective	18	12	Ranibizumab	4.2 ± 1.3	−71 *	-	−0.07	-
Kang *et al*., 2013 [156]	Retrospective	36	12	Ranibizumab	5.39 ± 0.50	-	22.2 at 3 months	-	25
			24		3.00 ± 0.53	-		-	-
			36		3.72 ± 1.01	294.2 ± 114.9		0.78 ± 0.53	19.4

*: From baseline.

### 10.4 Combination Therapy

Combination therapy is believed to be effective in the treatment of PCV because anti-VEGF may work synergistically with PDT in two ways: Firstly, by targeting the two components of PCV separately, combining its anti-angiogenic effect on the exudation from abnormal vascular networks with the angio-occlusive effect of PDT on polyps, and secondly by counteracting the upregulation of VEGF after PDT that results from choroidal hypoperfusion [183,184,185,186]. Lee *et al*., conducted a prospective randomized controlled trial that compared the two treatments in terms of their influence on aqueous humor levels of VEGF at three months after a single treatment [187]. VEGF levels were suppressed below the detection limit at one week, one month and in seven out of 12 patients at three months after treatment in the combination group. The other five patients had significantly lower VEGF levels at three months compared to baseline. In the PDT monotherapy group, however, VEGF levels rebounded to baseline levels at one month and three months after treatment. Again, the study was not powered adequately to assess clinically important endpoints like visual acuity and central foveal thickness, and did not show statistically significant differences between the two groups. Although the clinical applicability of this study is not immediately apparent, it suggests that adjunctive anti-VEGF treatment suppresses anti-VEGF levels up to three months and retreatment may not be necessary within this interval. Also, combination therapy may suppress recurrence from abnormal vascular networks by suppressing angiogenesis, although further studies are needed to elucidate the exact role of VEGF in polyp regression and recurrence. To further investigate the efficacy of combination therapy *versus* PDT monotherapy, Wang *et al*., conducted a systematic review and meta-analysis that analyzed two randomized controlled trials and nine high quality retrospective studies, including a total of 543 PCV cases [188]. No significant difference in visual acuity was found between PDT monotherapy and combination therapy at three and six months, but the combination group performed better in terms of mean visual acuity change at 12 months (weighted mean differences (WMD) 0.11, 95% CI 0.012, 0.21) and 24 months (WMD: 0.21; 95% CI: 0.054, 0.36; *p* = 0.008). Importantly, the study found that polyp regression, recurrence, central retinal thickness decrease and resolution of pigment epithelial detachment were not significantly different between the two groups. After 24 months, however, visual prognosis appeared to be more guarded [189,190,191,192,193,194,195,196]. To this end, Wong *et al*., in a meta-analysis comparing PDT monotherapy and PDT combination therapy, found significant visual gain in the combination group that persisted up to, but not beyond two years post treatment, while visual gain in the PDT monotherapy did not persist beyond one year [197]. Further, Gomi *et al*., found that eyes treated with PDT + bevacizumab combination therapy not only had better visual acuity up to 12 months post treatment than eyes treated with PDT monotherapy, combination therapy also resulted in fewer occurrences of subretinal hemorrhage within one month from PDT treatment (4.5% *vs*. 17.7%, *p =* 0.023) [198]. In summary, combination therapy appears to be a better treatment option for patients with PCV compared to PDT monotherapy. Whether combination therapy is also superior to anti-VEGF monotherapy could be answered by two randomized controlled trials that are currently in progress: the EVEREST II trial [199] and the PLANET trial [200].

### 10.5 Reduced Fluence PDT

Concerns with full fluence PDT, especially in patients who require multiple retreatments and in those who already have substantial chorioretinal atrophy have prompted the use of reduced fluence PDT for the treatment of PCV. Yamashita *et al*., prospectively reported favorable two-year visual outcome of 38 patients who underwent PDT with reduced fluence of 25 Jcm^−2^. Logmar visual acuity improved from a baseline of 0.43 to 0.28 at 12 months [201] and 0.29 at 24 months [202] (*p <* 0.001 and *p =* 0.001) after a mean number of 1.9 treatment sessions. A few studies have also found that reduced fluence PDT may be effectively combined with anti-VEGF [203,204,205,206,207].

Sakurai *et al*., retrospectively compared reduced fluence PDT in combination with ranibizumab and ranibizumab alone in 47 patients with PCV and found significant visual acuity improvement in the combination group (0.55 to 0.38 logMAR units, *p =* 0.041) but not the monotherapy group at 12 months [206]. Sagong *et al*., looked at bevacizumab in combination with reduced fluence PDT and found similar encouraging results, with improvement of logMAR visual acuity from 0.76 at baseline to 0.46 at month 12 (*p =* 0.002) [207]. None of these studies have directly compared reduced fluence with full fluence PDT but they do suggest that reduced fluence PDT, either alone or in combination, could be a viable treatment option at least in patients where full fluence PDT may pose significant concerns.

### 10.6. Radiotherapy

Stereotactic radiation therapy (SRT) is a form of external beam radiation therapy that was developed to address the problems posed by traditional external beam radiotherapy, specifically collateral damage to important structures adjacent to the intended treatment area. By focusing small doses of radiation from different angles that converge precisely at the treatment area, the maximum dose of radiation can be delivered to a small area while avoiding healthy adjacent tissue. While primarily used for the treatment of various malignant tumors, it has also been successfully used for the treatment of small intracranial arteriovenous malformations, where it works by damaging the endothelium with subsequent vascular occlusion from vessel wall enlargement [208]. The INTREPID study investigated the efficacy of SRT in the treatment of neovascular AMD, by delivering X-ray radiation through three points of entry in the inferior pars plana [209,210]. In this double masked, sham controlled randomized trial, patients who received a single dose of either 16-Gy or 24-Gy SRT required significantly fewer ranibizumab treatments over two years (mean 4.5 and 5.4 *vs.* 6.6, *p =* 0.008 and *p =* 0.09, respectively, *vs*. sham), with the best response where the choroidal new vessels fit within the treatment zone. However, the trial was unable to demonstrate a benefit in visual outcome compared to ranibizumab therapy alone (mean change in best corrected visual acuity from baseline: −10.0, −7.5 and −6.7 in the 16-Gy, 24-Gy and sham arms, respectively). Microvascular changes attributable to radiation were seen in 12% of patients but in only 1.3% was vision affected. Following this preliminary success, Introni *et al*., evaluated the efficacy of SRT in conjunction with ranibizumab in the treatment of 12 patients with macular PCV in a non-comparative, interventional study [211]. They found an 83.3% polyp regression rate at month 3 that persisted till month 12, and an improvement of 7.6 ETDRS letters at month 12 after a single dose of 16-Gy SRT and as needed ranibizumab. These results are comparable with those of PDT combination therapy described in other studies [188,197]. Epimacular Brachytherapy (EMB) is another form of radiotherapy that has been evaluated for the treatment of neovascular AMD, involving the placement of a radiation source adjacent to the active lesion following pars plana vitrectomy [212,213,214,215,216]. However, visual outcomes have been disappointing and there have been no studies of its efficacy in PCV.

### 10.7. Management of Submacular Hemorrhage

Submacular hemorrhage can occur in exudative AMD, particularly in PCV, with devastating effect on visual outcome if left untreated [110]. Tissue plasminogen activator (t-PA), an enzyme that promotes clot breakdown by catalyzing the conversion of plasminogen to plasmin, has been used with some success in the treatment of submacular hemorrhage associated with exudative AMD. T-PA can be administered into the subretinal space, in which case a vitrectomy with gas or air tamponade is performed, or injected intravitreally followed by pneumatic displacement with intravitreal gas injection. Neither technique has been shown to be superior or have a better safety profile than the other [217]. In both techniques, the aim is to displace the clot away from the fovea and hasten reabsorption of hemorrhage, thereby reducing blood-induced toxicity to the retina and RPE. Both methods can be combined with the use of anti-VEGF to treat the underlying cause of submacular hemorrhage and to target sub-RPE hemorrhage that is believed to be resistant to pneumatic displacement. However, anti-VEGF monotherapy alone may be effective for thin submacular hemorrhage [218,219,220,221,222], while avoiding complications of rhegmatogenous retinal detachment and choroidal hemorrhage that can occur with pneumatic displacement. Shin *et al*., compared ranibizumab/bevacizumab monotherapy with the combination therapy of ranibizumab/bevacizumab + pneumatic displacement in eyes with submacular hemorrhage of less than one month duration, secondary to CNV-AMD or PCV. Of note, reduction of central foveal thickness and visual improvement were more rapid in the combination therapy group at one-month post treatment, but no difference was found between the groups at six months. In addition, eyes with subretinal hemorrhage thicker than 450 µm had better visual outcome with combination therapy, but no difference was seen for submacular hemorrhage less than 450 µm. These results suggest that combination therapy may be more useful for patients who require faster visual improvement and in patients with thicker submacular hemorrhage.

### 10.8. Surgical Options and Retinal Pigment Epithelium Transplantation

In brief, because advances in treatment for AMD have so far been driven by pharmacological therapeutics, surgical treatment for AMD has been relegated to a complementary/experimental role. The Submacular surgery trials have shown that surgical excision of choroidal neovascular complexes alone offered no benefit over laser photocoagulation or observation alone, and were often complicated by retinal detachment and lens opacification [223,224]. Visual outcome was poor because RPE was often damaged and not replaced. In recognition of this limitation, surgical approaches that either transpose healthy retina overlying fibrotic RPE onto healthy RPE (macular translocation), or replace the damaged RPE with autologous/stem cell derived RPE transplants were developed. Macular translocation showed some visual benefit but was plagued with high rates of complications including proliferative vitreoretinopathy, macular hole, macular pucker, retinal detachment, torsional diplopia and recurrent choroidal neovascularization. Furthermore, RPE atrophy may progressively involve the new foveal region, thereby limiting the long term visual prognosis [225]. Autologous RPE transplantation can be achieved by harvesting RPE cells or RPE/choroid grafts from the patient’s peripheral retina. Problems with autologous RPE transplants include prolonged surgical time thus exposing the patient to higher rate of complications and insufficient RPE from the peripheral retina. Of late, there has been considerable interest in the use of stem cells (embryonic, induced pluripotent or multipotent) derived RPE which have been shown *in vivo* to improve visual function [226]. A phase 1/2 study of human embryonic stem cell derived RPE transplantation for atrophic AMD showed possible visual improvement with no evidence of rejection, abnormal proliferation, ocular or systemic adverse events [227].

## 11. Conclusion and Future Directions

Considerable research has been undertaken to understand Asian AMD, and PCV in particular. Yet many questions remain. First, the epidemiology of PCV has been largely limited to hospital-based studies, mainly due to the difficulty in diagnosing PCV from non-invasive imaging methods in population-based studies. Second, the pathogenesis of PCV is still unclear, with evidence that it may be part of a systemic vasculopathy. Potential therapeutic targets should be investigated, as guided by recent genetic studies and discovery of serum biomarkers, as well as how these may influence the outcomes of currently available pharmacological therapy. Third, a non-invasive, accurate imaging modality that can provide sufficient structural and functional information to replace ICGA is needed. Fourth, the optimal treatment for PCV requires further clarification, particularly with the introduction of aflibercept and possible benefits of reduced fluence PDT. Finally, stem cell based RPE transplantation may allow us to breach the final frontier in our battle with this blinding disease, by reversing what is currently irreversible damage to the RPE.
